# Effect of Diet and Dietary Components on the Composition of the Gut Microbiota

**DOI:** 10.3390/nu13082795

**Published:** 2021-08-15

**Authors:** Ashley Beam, Elizabeth Clinger, Lei Hao

**Affiliations:** 1 Department of Food and Nutrition, Indiana University of Pennsylvania, Indiana, PA 15705, USA; KSFW@iup.edu (A.B.); elizabethclinger24@gmail.com (E.C.); 2 Nutrition with Elizabeth, LLC, Brookville, PA 15825, USA; 3 Department of Nursing and Allied Health Professions, Indiana University of Pennsylvania, Indiana, PA 15705, USA

**Keywords:** diet, gut microbiota, chronic disease

## Abstract

Diet and dietary components have profound effects on the composition of the gut microbiota and are among the most important contributors to the alteration in bacterial flora. This review examines the effects the “Western”, “plant-based”, “high-fat”, “medical ketogenic”, and “Mediterranean” diets have on the composition of the gut microbiota in both mice and human subjects. We show that specific dietary components that are commonly found in the “plant-based” and “Mediterranean” diet play a role in shifting the microbial composition. This review further evaluates the bacterial metabolites that are associated with diet, and their role in systemic inflammation and metabolic endotoxemia. Furthermore, the associations between diet/dietary components and altering bacterial composition, may lead to potential therapeutic targets for type II diabetes, obesity, and inflammatory diseases.

## 1. Nutrition-Related Chronic Diseases

Data from 2017–2018 indicate that the prevalence of obesity increased to 42.4%, from a prevalence of 30.5% in 2000 [1]. Recent and recurring studies continue to show that being overweight and/or obese is a major risk factor for developing type II diabetes [1,2,3]. In fact, trends in the prevalence and incidence of type II diabetes and obesity mirror one another [1]. Currently, more than 34 million Americans have diabetes and 90–95% of those have type II diabetes. Of the 90–95% that have type II diabetes, 89% of them are either overweight or obese [1,2]. The main contributor to obesity, and potentially the development of type II diabetes, is consumption of a diet that is high in fat, high in sugar, and low in fiber [2,3]. The food that is consumed affects the bacterial composition within the gut microbiome, and the gut microbiome plays a vital role in food absorption, nutrient, and energy extraction, and low-grade inflammation, all of which have the potential to lead to obesity and type II diabetes [2,3].

## 2. Gut Microbiota Overview

There are 10 times the amount of microbial cells in the human gut than in the whole human body, totaling roughly 100 trillion microbes, representing as many as 5000 different species, and weighing approximately 2 kg [4]. Gut microbiota composition includes bacteria, viruses, fungi, and parasites [4]. Furthermore, the main species of bacteria include *Prevotella*, *Ruminococcus*, *Bacteroidetes*, and Firmicutes [5]. In the typical adult, Firmicutes are the most abundant, followed by *Bacteroidetes* and Actinobacteria [6]. Firmicutes can be broken down into *Clostridium*, *Ruminococcus*, and *Eubacterium*. The ratio between the bacterial species *Bacteroidetes* and Firmicutes has been shown to play an important role in health and disease [7]. Bacteria within the gut microbiome are involved in harvesting energy from food, balancing the beneficial and opportunistic bacterial composition, and manufacturing neurotransmitters, such as serotonin, enzymes, and vitamins. For instance, vitamin K, which is produced from bacteria, is involved in both immune and metabolic functions. As a result, when there is an imbalance in bacterial species, disease could result [4]. With diet noted to be one of the most powerful influences to alter the bacterial composition, a change in the diet composition can affect this ratio [4]. This review aims to further examine the role diet plays on the gut microbiome and its implication for disease, specifically obesity and type II diabetes.

## 3. Effect of Animal-Based Diet on Gut Microbiome

**Figure 1:**
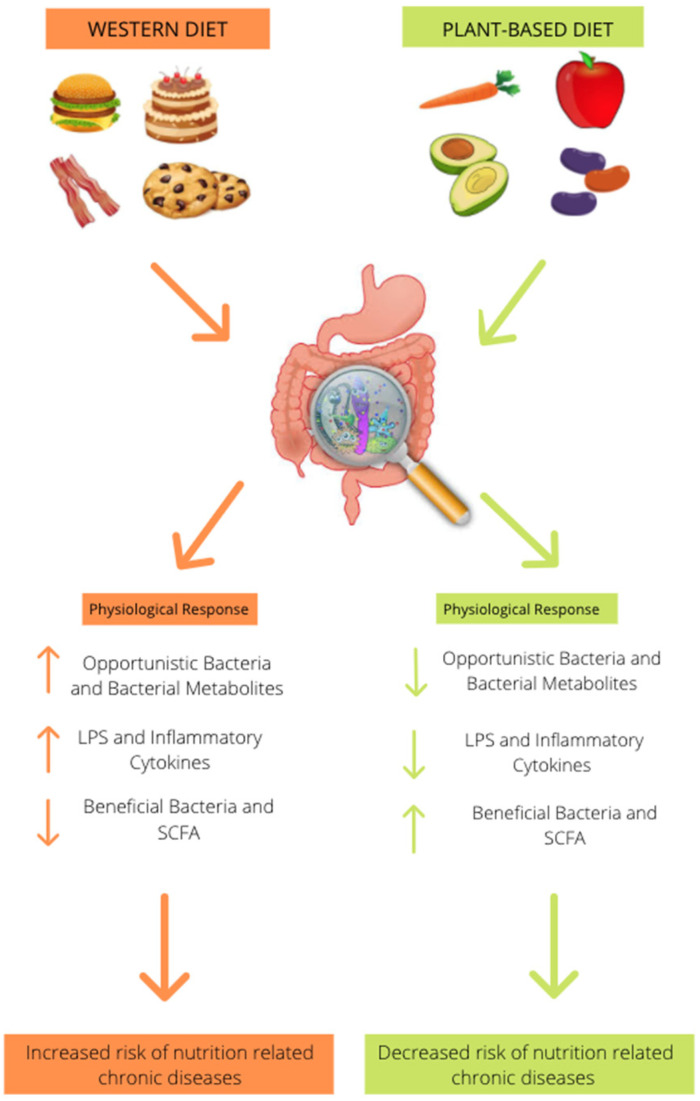
A traditional “Western diet” that is high in fat, high in processed sugar, and low in fiber results in an increase in Firmicutes, Proteobacteria, Mollicutes, *Bacteroides* spp., *Alistipes* spp., and *Bilophila* spp., Enterobacteriaceae, *Escherichia*, *Klebsiella*, and *Shigella* while decreasing the beneficial bacteria *Bacteroidetes*, *Prevotella*, *Lactobacillus* spp., *Roseburia* spp., *E. Rectale*, *Bacillus bifidus* and *Enterococcus* resulting in a reduction in SCFA production. It also increases LPS, TMAO, and inflammatory cytokines increasing risk for nutrition-related chronic diseases, obesity, and type II diabetes. Adherence to a plant-based diet that is rich in whole grains, fruits, and vegetables had inverse effects on the bacterial composition. It reduced opportunistic bacteria resulting in a reduction in LPS, TMAO, and inflammatory cytokines. It also increased the production of SCFAs, reducing inflammation and risk for obesity and type II diabetes.

Studies have proven that diet is a main contributor to the alteration in gut microbiome diversity, both in the short term and the long term. Recent documentation poses the idea that the bacterial composition, as a result of diet, can be linked to certain diseases, specifically diseases that arise due to chronic low-grade inflammation, such as type II diabetes [8,9,10,11,12]. A diet that is high in animal protein increases *Bacteroides* spp., *Alistipes* spp., and *Bilophila* spp., while it decreases the beneficial bacteria *Lactobacillus* spp., *Roseburia* spp., and *E. rectale*, (Figure 1) affecting the bacterial diversity in the gut microbiome [8,9,10,11,12]. A recent study that was conducted on mice, who were fed either a high-fat, high-sugar diet, or a low-fat, high-sugar diet, found that the mice who were fed the high-fat, high-sugar diet had a reduction in *Bacteroidetes*, and an increase in both Firmicutes and Mollicutes [8]. A study was conducted by Zhang et al., which found that when mice were fed a high-fat diet, there was a significant reduction in lactic acid and *Enterococcus* [13]. In another study, investigators conducted an experiment on mice, who were randomly assigned to either a normal chow diet or a high-fat diet, and found similar results, showing a change in gut microbiota composition. It compounded the findings of previous research, in which Proteobacteria and Firmicutes were found in abundance within the high-fat diet group. In addition, this study found that Enterobacteriaceae, *Escherichia*, *Klebsiella*, and *Shigella* were also found in higher levels in the high-fat diet group. Overall, evidence from these studies show a correlation between a high-fat diet and gut microbiota changes; *Bacteroidetes* were reduced and Firmicutes were increased (Figure 1) [14,15,16,17,18]. The typical “Western diet”, consisting of high-fat, high-sugar foods, has also been linked to chronic low-grade inflammation, metabolic disease, and obesity. Recent studies shown that a diet that is high in animal and saturated fats has the potential to alter the gut microbiota, by increasing lipopolysaccharides (LPS), increasing trimethylamine-N-oxide (TMAO), and decreasing short-chain fatty acids (SCFA) (Figure 1) [10,12]. The gut permeability to bacterial lipopolysaccharides (LPS) may be an important trigger for low-grade systemic inflammation; however, the mechanisms that allow LPS absorption are unclear, but could be related to an increased filtration of plasma LPS into the lymph, alongside fat absorption [8]. LPS are found on the outer membrane of Gram-negative bacteria, such as *Proteobacteria*, and serve as an endotoxin [19]. LPS are absorbed into the intestinal capillaries, to be transported with chylomicrons [8,20]. The increase in circulating LPS may be due to the increase in intestinal permeability, from the reduction in the expression of Zonulin occludens-1 (ZO-1), claudin, and occludin, which create the intestinal barrier [8]. The breakdown of the intestinal barrier results in LPS translocation, leading to inflammation and insulin resistance [8]. LPS activates Toll-like receptor 4 (TLR4) [21]. TLR4, which is present on most cells and also on macrophages, recognizes pathogen-associated molecular patterns (PAMP) [21]. The binding of LPS to TLR4 causes a signaling cascade via cytokine expression, inducing the inflammatory response [21]. Chronic low-grade inflammation via cytokine expression has been linked to insulin resistance, leading to type II diabetes [8,21]. A study conducted by Cani et al., to examine the role that LPS plays on metabolic endotoxemia in obesity and insulin resistance, found that that a 4-week high-fat diet chronically increased plasma LPS concentration was two-to-three times the threshold value for metabolic endotoxemia [8]. A high-fat diet also increased the proportion of an LPS-containing microbiota in the gut (Figure 1) [8]. Within this study, metabolic endotoxemia was induced for 4 weeks in mice, through continuous subcutaneous infusion of LPS [8]. They recorded an increase in fasted glycemia and insulinemia, and an increase in adipose tissue weight gain [8]. In addition, adipose tissue F4/80-positive cells, inflammatory markers, and liver triglyceride levels, were increased [8]. Lastly, they found that liver insulin resistance was detected in LPS-infused mice, concluding that LPS could be a contributing factor to insulin resistance and the onset of type 2 diabetes [8]. To further investigate the effects of a high-fat diet, C57BL/6J- and TLR4-deficient C57BL/10ScNJ mice were maintained on either a low-fat diet or a high-fat diet for 8 weeks, to examine if a high-fat diet induces gut microbiota inflammation [22]. This study, by Kim et al., provided evidence that a high-fat diet may increase inflammation and circulating proinflammatory cytokines (Figure 1), via the TLR4 signaling pathway, activating necrosis factor in the colon [22]. The high-fat diet also reduced the expression of claudin-1 and occludin, as previously mentioned, resulting in LPS translocation and inflammation [22]. This study, by Kim et al., provided evidence that a high-fat diet may increase inflammation via the TLR4 signaling pathway [22]. The changes in the gut microbiota that were observed may also be a result of the increase in secondary bile acids, ammonia, phenolics, hydrogen sulfide, and choline, found in red meat, processed meat, and animal fat [23,24]. However, the ketogenic diet has been shown to have an inverse effect on the gut microbiome that could potentially combat inflammation and decrease insulin resistance [25]. A study that was conducted on a murine model, found that, unlike the traditional high-fat diet, the ketogenic diet decreased Firmicutes and increased *Bacteroidetes* [25]. Additionally, Olson et al. conducted a study on mice, in which one group was fed the ketogenic diet and the other group was fed a normal chow. Researchers found that the ketogenic diet increased *A. muciniphila*, *Parabacteroides* spp., while decreasing alpha diversity [26]. Another study that was conducted on mice, found similar results, in which the ketogenic diet increased *Akkermansia muciniphila* and *Lactobacillus*, while it reduced the inflammatory bacteria *Desulfovibrio* and *Turicibacter* [27]. When conducted on humans, similar results were found. A randomized, double-blind, crossover study, on the modified Mediterranean ketogenic diet (MMKD) versus the American Heart Association diet, found that *Bifidobacterium* and *Lachnobacterium* were reduced on the MMKD, and *Akkermansia*, *Slackia*, and *Christensenellaceae* were increased [28]. They also found that MMKD decreased fecal lactate and acetate, while increasing propionate and butyrate [28]. Another study also examined the effect that the ketogenic diet has on the gut microbiome. For this study, C57BL/6J mice followed three different diets for 3 weeks, including a low-fat diet, high-fat diet, and ketogenic diet [29]. The findings indicated that a ketogenic diet decreases the relative abundance of *Actinobacteria*, *Lactobacillus*, and *Bifidobacterium* [29]. Researchers further examined the phenomenon of *Bifidobacterium* and *Lactobacillus* decreasing, and connected it to the production of beta-hydroxybutyrate from ketone bodies that were produced when the body was in ketosis [29]. They further explored this idea, by providing the high-fat diet group with a synthetic ketone ester to mimic beta hydroxybutyrate [29]. This resulted in a significant increase in beta-hydroxybutyric acid (BHB) levels and a significant decrease in both *Bifidobacterium* and *Lactobacillus* [29]. In regards to the ketogenic diet reducing inflammation, a study conducted by Ang et al., on germ-free mice, showed that when given a high-fat diet there was an increase in *Bifidobacteria*, resulting in the induction of proinflammatory Th17 cells [29]. However, when colonized with *B. adolescentis* and supplemented with a ketogenic diet, induction was prevented [29]. Also, when the mice were fed the ketogenic diet, they had a reduction in circulating Th17 cells [29]. To further investigate this, the mice were fed a high-fat diet and supplemented with the ketone ester, which resulted in a reduction in *Bifidobacteria* and Th17, showing that the ketogenic diet has the potential to reduce Th17 cells and thus inflammation within the gut microbiome. This finding could be a potential therapeutic target to combat the inflammatory response and autoimmune disease [29].

## 4. Effect of Plant-Based Diet on Gut Microbiome

There are the following three enterotypes that are prominent in the human microbiome: *Bacteroides*, *Ruminococcus*, and *Prevotella* [5]. However, some other studies have indicated that enterotype distribution may be continuous, rather than discrete [30]. There has been a significant amount of research, over the past decade, regarding how different diets affect enterotype distribution within the host’s gut microbiome [31]. Research indicates that high levels of *Prevotella* species are linked to plant-based dietary habits [32,33,34]. To further the research, a study that was conducted on children from Burkina Faso and Italy, examined the effect that diet has on bacterial composition [35]. The European children consumed a diet that was similar to that of the Western diet, being low in fiber, while the Burkina Faso children (African children) had a diet that was rich in fiber and resistant starch [35]. Researchers found that the Burkina Faso children had a microbiome that was enriched with *Bacteroidetes*, and genus *Prevotella* and *Xylanibacter*, while being depleted of Firmicutes [35]. The African children also had significantly more SCFA production when compared to the European children [35]. An animal study, using MiR-146a-deficient mice, examined the impact that a plant-based diet that is rich in miR-146a would have on the microbial communities [36]. This study showed that the microbiome of the mice fed the plant-based diet was significantly different from the microbiome of the mice fed the control diet, allowing the researchers to conclude that an increase in dietary fiber results in a shift of microbial communities [36]. For the mice consuming the plant-based diet, they found that when the mice switched from a chow diet to a plant-based diet, there was a significant increase in *Bacteroides* and *Alloprevotella*, and a decrease in Porphyromonadaceae and Erysipelotrichaceae [36]. To further the idea of a high-fiber diet altering the bacterial composition, a human diet intervention study was conducted, to examine the bacterial composition based on what the participants reported as their typical diet [31]. Ninety-eight subjects, who identified as vegetarian, showed enrichment of *Prevotella*, while those who consumed a typical Western diet had a microbiome environment that was enriched with *Bacteroides* [31]. Researchers also noted that when ten of the subjects switched diets, their microbiome composition was altered within 24 h of consumption of the other diet [31,32]. A similar study showed results that aligned; however, it was completed with Thai subjects [37]. They also found that the vegetarian subjects had microbiomes that were enriched with *Prevotella* when compared to non-vegetarians [37]. A confounding study that compared the bacterial DNA from the fecal samples of 20 vegans, 11 lacto-vegetarians, and 29 omnivores, found that there is in fact an association that exists between diet type and bacterial composition, but in this study, both the vegetarian and vegan groups were associated with a higher ratio of *Bacteroides*-to-*Prevotella* when compared to the omnivore group [38]. Low-fat, high-fiber diets have the ability to alter the microbial intestinal composition in a positive manner, by shifting the microbiome environment towards the beneficial bacteria *Prevotella* and *Bacteroides*, while shifting away from Firmicutes (Figure 1) [38].

## 5. Effect of Mediterranean Diet on Gut Microbiota

The Mediterranean diet is plant focused, high in fiber and omega-3 fatty acids, and low in animal protein and saturated fat. It has been shown that adherence to this diet was found to be associated with increased levels of SCFA, *Prevotella*, and fiber-degrading Firmicutes [39]. In this specific study, researchers also found that the ratio of *Prevotella*-to-*Bacteroides* was greater in those who adhere to the Mediterranean diet, indicating that a diet that is high in natural fiber and resistant starch positively alters the bacterial composition of human subjects [39]. Additionally, a similar study was conducted, utilizing a food frequency questionnaire and a microbiota composition analysis. Upon completion, it was found that low adherence to the diet resulted in a higher Firmicutes-to-*Bacteroidetes* ratio [40]. When the subjects had better adherence to the Mediterranean diet, they had a greater presence of *Bacteroidetes*, higher counts of *Bifidobacteria*, and higher levels of SCFA [40]. Similarly, Mitsou et al. found that high adherence to the Mediterranean diet correlated with lower *Escherichia coli* counts, a higher *Bifidobacteria*:*E. coli* ratio, increased levels of Candida albicans, and a greater amount of SCFA acetate [41]. Nagpal et al. conducted a study on mice, to further analyze the gut microbiome after adhering to either the typical Western diet or Mediterranean diet [42]. The Western diet consisted of lard, beef tallow, butter, egg, cholesterol, casein, lactalbumin, dextrin, high-fructose corn syrup, and sucrose; while the Mediterranean diet was comprised of fish oil, olive oil, fish meal, butter, egg, black and garbanzo bean flour, wheat flour, V-8 juice, fruit puree, and sucrose [42]. They found that the microbiome of the study participants consuming the Mediterranean diet was significantly more diverse when compared to the microbiome of participants consuming the Western diet [42]. They also had a higher abundance of *Lactobacillus*, *Clostridium*, *Faecalibacterium*, and *Oscillospira*, and a lower abundance of *Ruminococcus* and *Coprococcus* [42]. These results are consistent with a human study that was conducted by Pagliai et al., in which they found that, after a 3-month Mediterranean diet intervention, the subjects had a significant change in their gut microbiome composition, and had an abundance of *Enterorhabdus*, *Lachnoclostridium,* and *Parabacteroides*, with increased production of SCFAs [42]. The diet also resulted in a reduction in the inflammatory cytokines VEGF, MCP-1, IL-17, IP-10, and IL-12 [43]. Another study that investigated the effects of the Mediterranean diet on inflammatory markers was conducted by Ghosh et al., in which 612 non-frail or pre-frail subjects, across five European countries (UK, France, Netherlands, Italy, and Poland), were analyzed before and after a 12-month long Mediterranean diet intervention [44]. Adherence to the diet was negatively associated with the inflammatory markers CRP, IL-17, and IL-2 [44]. It also resulted in positive levels of anti-inflammatory cytokines IL-10 [44]. Adherence to the Mediterranean diet had positive health associations, including the production of short-chain fatty acids (SCFAs) and anti-inflammatory properties reducing the risk of chronic inflammatory diseases, such as type II diabetes.

## 6. Fiber and the Gut Microbiome

### 6.1. Fiber

Dietary fiber refers to non-digestible carbohydrates and lignin that are intact and intrinsic in plants. Dietary fiber differs in chemical structure, water solubility, viscosity, and fermentability [45]. Dietary fibers are carbohydrate polymers that contain three or more monomeric units that are resistant to digestive enzymes, and are not hydrolyzed or absorbed in the small intestine [46]. They are further broken down into the groups soluble fiber and insoluble fiber. Soluble fibers have been of much interest, due to them being metabolized by the gut bacteria and producing short-chain fatty acids (SCFA) [46]. The bacterial species that are the most respondent to dietary fiber are those who belong to Firmicutes and Actinobacteria [46]. Over the past few decades, there has been much discussion about plant food components and their influence on disease; however, more recently, they are discovering their effects on the gut microbiota. A meta-analysis showed that dietary fiber intervention, particularly involving fructans and galacto-oligosaccharides, results in a higher abundance of *Bifidobacterium* and *Lactobacillus* spp., but does not change the α-diversity [47]. High-fiber diets have been linked to an increase in SCFA production within the gut [48,49]. A recent review further examined this idea of dietary fiber and SCFA production. Dietary fibers escape digestion and are metabolized by the bacterial flora within the gut microbiome, producing the SCFAs [46,50]. When dietary fiber was in short supply, SCFA production was reduced [46,50]. Not only is SCFA production reduced when fiber intake is low, but it also causes the gut microbiota to utilize less-favorable substrates, such as amino acids and host mucins, for energy [46,50]. A specific study that consisted of 178 elderly individuals, who consumed either a low-fiber diet or a high-fiber diet, found that the high-fiber diet group had a higher proportion of SCFA butyrate, acetate, and propionate compared to those who were fed low-fiber diets [48]. Similarly, in a randomized controlled trial, where one group was given a high-fiber diet with probiotics and the other group received a control diet, they found that there was an increase in SCFA production in the high-fiber group, specifically acetate and butyrate [48]. Another randomized-controlled pilot study, of 29 overweight and obese volunteers, investigated the effects of eating fiber- and phytochemical-rich, stabilized rice bran or cooked navy bean powder on the gut microbiota [51]. The subjects who received the rice bran had a significant decrease in the Firmicutes-to-*Bacteroidetes* ratio [51]. They also had a significant increase in the SCFAs propionate and acetate [51]. Kaczmarek et al. conducted a study on broccoli consumption, to examine the effect of fiber on the gut [52]. Researchers found that broccoli consumption decreased the relative abundance of Firmicutes by 9% compared to the control, increased the relative abundance of *Bacteroidetes* by 10% compared to the control, and increased *Bacteroides* by 8% relative to the control [52]. An increase in fiber increases SCFA production, which reduces inflammation [53]. A specific study that further examined this idea was conducted by Kopf et al. The purpose of this study was to determine the impact of increasing the intake of either whole grains or fruits and vegetables on inflammatory markers and gut microbiota composition [53]. It was a randomized, parallel feeding trial, involving overweight or obese subjects with low intakes of whole grains and fruits and vegetables [53]. The subjects were randomized into the following three groups: whole grain, fruit/vegetable, or a control group, which consumed a diet that consisted of refined grains [53]. Inflammatory markers were measured and a stool analysis was collected, to analyze the microbiota composition [53]. They found a significant decrease in LPS for both the whole-grain diet group and the fruit/vegetable-diet group [53]. The fruit/vegetable diet resulted in a significant change in IL-6, and the whole-grain diet resulted in a significant decrease in TNF [53]. Another study that examined inflammatory markers within the gut was conducted by Jang et al., where the subjects were treated with doenjang, a soybean paste that is high in fiber and antioxidants [54]. They found that, similarly to the whole-grain diet in the previous study, it significantly decreased LPS and TNF [54].

### 6.2. Prebiotic Inulin

Another commonly examined food component is prebiotic inulin. A study that was conducted by Birkeland et al., in May 2020, examined the effect that prebiotic inulin-type fructans had on the fecal bacteria and SCFA production in patients with type II diabetes, and found that treatment with the inulin-type fructans resulted in moderate changes in the microbial composition of type II diabetics, with the bifidogenic effect being most prominent on *Bifidobacterium adolescentis* [55]. In addition, the test subjects who were given the inulin-type fructan, had significantly higher SCFA acetic acid and propionic acid [55]. Another recent double-blind, placebo-controlled, crossover study, with 32 adults, examined the prebiotic action of inulin [56]. In this study, over a period of 3 weeks, two groups consumed 10 g/day of either very-long-chain inulin that was extracted from globe artichoke, or a placebo that was composed of maltodextrin [56]. They found that the very-long-chain inulin consumers had greater bifidogenesis, increased lactobacilli, and a decreased *Bacteroides–Prevotella* ratio [56]. Kleessen et al. conducted another double-blind, randomized, placebo-controlled study, in which 15 volunteers either consumed vegetable snack bars that had 7.7 g inulin (sourced from either Jerusalem artichoke, chicory, or cereal), or a placebo [57]. Consuming the inulin resulted in a bifidogenic effect that was similar to the results from the previously mentioned study [54]. It also showed a significant reduction in the *Bacteroides–Prevotella* ratio, which was consistent with the results from previous studies [57]. To further investigate this, another randomized, controlled, double-blind crossover study, by Birkeland et al., found that supplementation of inulin-type fructan resulted in a significant increase in bifidogenesis and total SCFA production, specifically acetic and propionic acid [55].

### 6.3. Resistant Starch

Resistant starch has long been an established food component that has received considerable attention for positively impacting the gut microbiota. A seminal study that was conducted on the intestinal flora of rats, in 1997, examined the long-term effects of a diet that was supplemented with resistant starch 1 (RS1 from native potato starch), or supplemented with resistant starch 2 (RS2 from modified potato starch) [58]. Both the RS1- and RS2-fed rats had an increase in *Bifidobacterium* spp. [58]. The RS2 group, however, also had an enhanced composition of lactobacilli, streptococci, and Enterobacteriaceae [58]. They also found the production of SCFA to be increased in both the groups when compared with the control group [58]. A similarly conducted study on rats given resistant potato starch, found results that were consistent with previous studies; in particular, there was an enhancement in SCFA butyrate production [59]. To further examine this, a human study, conducted in 2010, assessed the SCFA levels in subjects who were supplemented with a low-fiber control diet or a diet that was composed of 30 g wheat bran fiber, RS2, or RS3 [60]. They discovered that, similarly to the results from previous studies that were conducted on rats, the butyrate-to-SCFA ratio was significantly increased by the resistant starch diet [60]. Another randomized, crossover trial, examining diet interventions on 46 human volunteers, was conducted by Abell et al., in 2008 [61]. One group was given a diet that was high in RS2 and the other group was given a diet that was low in RS2 [61]. Researchers found that the RS2-diet group had a microbiota that was enriched in *Ruminococcus bromii*, *F. prausnitzii*, and *E. rectale* [61]. They also found that *E. rectale* was positively correlated with the production of SCFAs, mainly butyrate [61]. Similarly, another placebo-controlled, double-blind crossover trial that examined the effect of RS2 on the composition of the gut microbiota, found that RS2 significantly increased *R. bromii* and *E. rectale* [61].

## 7. Phytochemicals and Their Effect on the Gut Microbiome

### 7.1. Polyphenols

Polyphenols have been popularized in discussion, especially for the anti-cancer properties that they possess; however, recently they have been shown to have positive effects on the gut microbiome. An example of this research is a study in which the subjects consumed a wild blueberry powder drink for six weeks, to examine the polyphenol effect of blueberries on the gut [62]. The subjects who adhered to the consumption of the drink for six weeks had an increase in *Bifidobacterium* and *Lactobacillus* [62]. A similar study, by Queipo-Ortuño et al., examined the influence that red wine polyphenols have on the gut microbiota [63]. Researchers found similar results, in that the consumption of the red wine was associated with an increase in *Bifidobacteria* [63]. They also found that there was an increase in the beneficial bacteria *Bacteroides* and *Prevotella* [63]. Another randomized, crossover, controlled intervention study, conducted on obese and metabolic syndrome subjects, was conducted to further examine polyphenol’s role in the gut microbiome [64]. The subjects consumed red wine and dealcoholized red wine, over a 30-day period for each [64]. In the metabolic syndrome patients, red wine polyphenols significantly increased the number of fecal *Bifidobacteria*, *Lactobacillus*, *Faecalibacterium prausnitzii*, and *Roseburia* [64]. They also resulted in less production of the Gram-negative bacteria *Escherichia coli* and *Enterobacter cloacae* [64]. A similar study found consistent results, in which *Bifidobacterium* and *Prevotella* amounts were significantly increased by red wine, and correlated negatively with LPS concentrations [65]. In another study, after the red wine consumption period, there was an increase observed in *Enterococcus*, *Prevotella*, *Bacteroides*, *Bifidobacterium*, *rectale* group, *B. uniformis*, and *Eggerthella* species group [66].

### 7.2. Flavonoid

Flavonoids are present in fruits, vegetables, legumes, nuts, and seeds. Flavonoids and their metabolites have been shown to exhibit positive gut-modulating properties, specifically on SCFA production and LPS reduction. A recent study examined the effects of proanthocyanin on the gut flora [67]. Researchers found that the proanthocyanin-rich extract from grape seeds significantly increased *Bifidobacterium* spp., while decreasing Enterobacteriaceae [67]. This is also consistent with another study that found that flavonoids increase *Bifidobacterium* and *Lactobacillus* [68]. Utilizing batch culture fermentation, Molan et al. found consistent results with the above study, in which anthocyanins significantly enhanced *Lactobacillus*, *Enterococcus* spp., and *Bifidobacterium* spp. [68]. Another batch culture fermentation study, conducted by Hidalgo et al., found that flavanol-3-ol monomers promoted the growth of *Clostridium coccoides–eubacterium rectale*, which has the potential to produce large amounts of the SCFA butyrate [69]. Also, the monomer (+)− catechin increases the growth of *Lactobacillus–enterococcus* spp., *Bifidobacterium* spp., and *Escherichia coli* [69]. Interestingly, a study that was conducted to examine the effects of flavonoids on insulin resistance, found that the subjects who consumed a flavonoid-rich cranberry extract had a reduction in inflammation, by modulating the specific bacteria *Akkermansia muciniphila* [70]. To advance the idea of flavonoids reducing inflammation, other studies were examined. A study that was conducted on human subjects, who were given polyphenol-rich mango, found a reduction in endotoxin LPS, and an increased production of SCFA [71]. A similar study, where subjects consumed pomegranate extract containing both flavonoids and polyphenols, for 3 weeks, also found there to be a reduction in LPS, by modulating *Faecalibacterium*, *Odoribacter*, and *Parvimonas* [72].

## 8. Bacterial Metabolites Effect on Inflammation and Metabolic Endotoxemia

### 8.1. Short-Chain Fatty Acids (SCFA)

SCFA are bacterial metabolites derived as a result of fermentation of dietary fibers by bacterial flora within the gut microbiome [50]. It has been established that dietary fiber promotes weight loss and an improvement in glycemic control; however, several recent studies have been conducted, to examine the relationship between dietary fiber fermentation, the production of SCFA, and improved metabolism [50]. There has also been significant research on low-grade inflammation potentially causing insulin resistance and type II diabetes. More recently, there has been research on the effects that SCFA have on the gut microbiome, and their role in reducing inflammation, improving insulin resistance, and promoting satiety [50]. A study that was conducted by Chambers et al., in 2015, found that when human subjects were given an inulin propionate ester, they had a significant increase in postprandial GLP-1 and PYY, and reduced calorie intake [73]. When given long term, it resulted in a significant reduction in weight [73]. Similarly, PYY and GLP-1 were increased by intravenous perfusions of acetate [74]. Metabolic endotoxemia may result from chronic low-grade inflammation [75]. The balance between Treg lymphocytes, which have anti-inflammatory properties, and Th17 cells, which are pro-inflammatory, is vital for a proper inflammatory response. This is prominently modulated by the gut microbiota, specifically by SCFA [75]. SCFA lead to increased levels of both IL-10 and Treg cells, resulting in inflammation reduction [76]. IL-10 is released following the recognition of polysaccharide A by plasma dendritic cells [77]. Polysaccharide A is produced by the SCFA Bacteroidales, Erysipelotrichales, Clostridiales, and Bacillales [77]. SCFA also exhibit anti-inflammatory effects through inhibiting NF-B, by binding to the G-protein-coupled receptors 43 and 41 (GPR43 and GPR41) [78]. This interaction promotes downstream signaling and helps to regulate hypoxia-inducible factors, promoting intestinal integrity, and prevents the translocation of LPS combating inflammation [79]. Additionally, SCFA promote the activation of histone acetyltransferase and the inhibition of histone deacetylase enzymes, promoting anti-inflammatory phenotypes in the gut microbiome [80]. Not only do they inhibit NF-B, resulting in decreased inflammation, they also engage in the epigenetic regulation of inflammation, through free fatty acid receptors (FFARs) [81]. In terms of insulin sensitivity, SCFA, more recently, have been found to have a positive effect on glucose homeostasis [82,83]. A recent study, conducted on FFAR2- or FFAR3-deficient mice, found that the mice had low levels of circulating GLP-1 and impaired glucose tolerance, mainly due to the idea that GLP-1 promotes insulin sensitivity and satiety [82,83]. This study also found that SCFA had the ability to inhibit insulin-stimulated lipid accumulation in adipocytes, via FFAR2 signaling, allowing them to conclude that SCFA play a vital role in glucose homeostasis [82,83]. Additionally, acetate, propionate, and butyrate have been recognized as ligands of FFAR2 and FFAR3 [84,85]. The activation of FFAR2 in adipocytes causes the release of leptin and the secretion of peptide YY (PYY) [84,85]. A similar study that was conducted on overweight women, who were infused with SCFA acetate, found results that were consistent with previous studies [74]. Researchers also found that SCFA play a role in modulating satiety [74]. They advanced this hypothesis by analyzing GLP-1 and PYY, and found that when infused with acetate, there was an increase in the levels of both proteins in circulation, resulting in reduced appetite [74]. Acetate, along with propionate and butyrate, has also been shown, in multiple studies, to regulate hepatic lipid and glucose homeostasis [86,87]. Not only do SCFA play a role in glucose homeostasis in the liver, but also in the intestines [88]. Butyrate and propionate have been shown to induce intestinal gluconeogenesis, improving both peripheral glucose production and insulin sensitivity [88]. SCFA play a vital role in reducing intestinal inflammation and promoting glucose homeostasis; however, further research on human subjects needs to be conducted to confirm their role.

### 8.2. Bile Acids

Bile acids (BA) are secreted into the gut lumen, in the presence of fat [88]. There are two main types of primary bile acids, including (1) cholic acid and (2) chenodeoxycholic acid [88]. Primary bile acids are transformed by the gut microbiota, and they interact with farnesoid X receptor (FXR) and the G-protein-coupled bile acid receptor 1/TGR-5 [89]. Bile acids have metabolic effects, through farnesoid X receptor (FXR) and Takeda-G-protein-receptor-5 (TGR5) [90]. The activation of FXR and TGR5 promotes glycogen synthesis and insulin sensitivity in the liver, increases insulin secretion by the pancreas, and mediates satiety in the brain [89]. The regular consumption of animal fat promotes the production of taurocholic acid (TCA) [90]. TCA favors *Bilophila wadsworthia*, which is known to increase intestinal permeability, resulting in bacterial translocation [90]. This alteration in the microbiome could result in the impairment of bile acid absorption [90]. As a result, FXR and FGF19 expressions are decreased, causing an imbalance of BA [90]. The imbalance of BA plays a role in low-grade chronic intestinal inflammation [90].

### 8.3. Tryptophan

Indole derivatives and tryptamine have vital roles in the homeostasis of epithelial and immune cells in the gut [91]. These compounds are formed through tryptophan metabolism within the gut microbiome [91]. These metabolites have the potential to promote Th17 reprogramming to Treg cells, resulting in a decrease in inflammation [92]. However, when the host has an alteration in the gut bacterial composition, likely due to diet, it could cause a defect in the production of the aryl hydrocarbon receptor ligand indole-3-propionic acid [91]. The defect of this ligand leads to a decrease in the secretion of GLP-1 and IL-22, causing a break in intestinal permeability, resulting in LPS translocation and thus inflammation [90].

### 8.4. Trimethylamine (Tma/Tmao)

The typical Western diet is composed of a high consumption of red and processed meat, containing high levels of carnitine and choline, both of which are precursors for the gut bacteria to produce trimethylamine (TMA) [93]. TMA, created mainly by the gut bacteria Firmicutes and proteobacteria, is transported to the liver, to be converted into trimethylamine N-oxide (TMAO) from the enzyme flavin-containing monooxygenase 3 (FMO3) [93]. TMAO has been linked to inflammation and is shown to be associated with type II diabetes and obesity [94]. A study, conducted by Schugar et al., further investigated the relationship of the fasting plasma levels of choline or TMAO with type II diabetes risk, in two independent cohorts [94]. They found that the plasma concentrations of TMAO were significantly increased in the subjects who had type II diabetes [94]. In addition, they examined obesity-related traits and circulating TMAO levels in mice who were fed an obesogenic high-fat/high-sucrose diet, similar to that of the Western diet [94]. In regards to this study, researchers found that the plasma levels of TMAO were positively associated with fat mass and body weight [94]. To advance this idea, they wanted to determine if the expression of FMO3 was expressed in a different manner for obese and overweight individuals. Utilizing a random sample of 770 subjects, they found that FMO3 was positively correlated with BMI and waist-to-hip ratio [94]. They also found that it was negatively correlated with insulin sensitivity [94]. High-fat diets change the composition of the gut microbiome, increasing the relative proportion of Firmicutes, which are TMA-producing organisms, and further increasing systemic inflammation and insulin resistance [95].

## 9. What’s Next

Over the past decade, there has been extensive research about the association between body mass index, bacterial composition, diet, and their influence in chronic disease. Evidence indicates that there is a shift in the beneficial bacteria, *Prevotella* and *Bacteroides*, in the obese population, due to adherence to a high-fat, high-sugar, low-fiber diet. This shift in bacteria, from a poor diet, could lead to metabolic syndrome and type II diabetes, due to chronic low-grade inflammation. *Prevotella*, associated with plant-based diets, has been shown to have the most potent anti-inflammatory effects, followed by *Bacteroides*, which has a lesser effect. The current research provides evidence of the vital role that diet plays in the bacterial composition within the gut microbiome (Figure 1). Existing research shows that the adoption of a plant-based diet, as a therapeutic diet intervention, has beneficial effects on the host’s microbiome, helping to reduce inflammation, improve insulin sensitivity, and promote optimal energy balance, which could further lead to combating chronic diseases that are associated with low-grade inflammation. Due to the complexity of the human race, further studies need to be conducted to identify and understand how the plant-based diet affects the gut microbiome for all populations.

## Data Availability

Not applicable.
